# The effect of sexual orientation on voice acoustic properties

**DOI:** 10.3389/fpsyg.2024.1412372

**Published:** 2024-08-07

**Authors:** Luke Holmes, Gerulf Rieger, Silke Paulmann

**Affiliations:** Department of Psychology, University of Essex, Colchester, United Kingdom

**Keywords:** voice, acoustics, formants, sexual orientation, sexual identity

## Abstract

**Introduction:**

Previous research has investigated sexual orientation differences in the acoustic properties of individuals’ voices, often theorizing that homosexuals of both sexes would have voice properties mirroring those of heterosexuals of the opposite sex. Findings were mixed, but many of these studies have methodological limitations including small sample sizes, use of recited passages instead of natural speech, or grouping bisexual and homosexual participants together for analyses.

**Methods:**

To address these shortcomings, the present study examined a wide range of acoustic properties in the natural voices of 142 men and 175 women of varying sexual orientations, with sexual orientation treated as a continuous variable throughout.

**Results:**

Homosexual men had less breathy voices (as indicated by a lower harmonics-to-noise ratio) and, contrary to our prediction, a lower voice pitch and narrower pitch range than heterosexual men. Homosexual women had lower F4 formant frequency (vocal tract resonance or so-called overtone) in overall vowel production, and rougher voices (measured via jitter and spectral tilt) than heterosexual women. For those sexual orientation differences that were statistically significant, bisexuals were in-between heterosexuals and homosexuals. No sexual orientation differences were found in formants F1–F3, cepstral peak prominence, shimmer, or speech rate in either sex.

**Discussion:**

Recommendations for future “natural voice” investigations are outlined.

## Introduction

Homosexual individuals differ, on average, from heterosexual individuals on a wide range of measures: These include physical traits such as facial structure (Skorska et al., 2015; Wang and Kosinski, 2018), body size (Bogaert, 2003), and weight (Bogaert and Friesen, 2002; Laska et al., 2015), as well as psychological traits such as preferred hobbies and occupations (Lippa, 2010, 2020), personality traits (Lippa, 2008b), and gender-typed behavior in both childhood and adulthood (Bailey and Zucker, 1995; Rieger et al., 2008, 2010). On average, homosexual men are more feminine than heterosexual men, and homosexual women are more masculine than heterosexual women—a pattern known as gender nonconformity (Lippa, 2008a; Baams et al., 2013; Swift-Gallant et al., 2017; Rieger et al., 2020a). When bisexual individuals were studied, they appeared to be in-between heterosexual and homosexual with respect to masculinity and femininity (Rieger et al., 2020b).

Another difference which may exist between homosexual and heterosexual individuals of both sexes is in their voice properties. One proposal is that homosexuals of both sexes display vocal patterns more in line with the other sex (Suire et al., 2020). That is, homosexual men are expected to display more female-typical speech patterns, and homosexual women are expected to display more male-typical speech patterns, when compared to heterosexual members of the same sex. In accordance with this proposal, Gaudio (1994) suggested that homosexual males would have increased pitch range and variability when compared to heterosexual males. Zwicky (1997) polled a group of linguists on what they believed the differences might be between homosexual and heterosexual male speech, and their suggestions included wider pitch range and variability, higher pitch in general, and that homosexual males might speak in a “breathier” voice. In women, Moonwomon-Baird (1997) found that two homosexual women had a lower voice pitch and reduced pitch range and variability compared to two heterosexual women, but did not conduct statistical tests to confirm the findings.

More systematic research on this topic has yielded mixed results. One obvious point of focus is pitch: Given that there is a striking difference in mean fundamental frequency (F0, perceived as overall pitch) between most men and women (Aung and Puts, 2020), a possible sexual orientation difference would be that homosexual men have higher F0, and homosexual women lower F0, compared to heterosexual members of the same sex. A similar prediction could be made about F0 range, where the consensus is that men use a narrower pitch range than women (Gilmore et al., 1992; Waksler, 2001; Smyth et al., 2003). However, several studies have investigated these cues, and yielded mixed findings: Munson et al. (2006) did not find evidence that mean pitch, pitch range, or spectral tilt, also known as spectral slope (i.e., balance between high/low frequency energy present in the sound, associated with vocal noise/breathiness) differed by sexual orientation in 44 male and 44 female speakers. Other investigations on mean pitch alone also did not find expected sexual orientation differences in samples of 8, 125, 25, and 160 participants, respectively (Gaudio, 1994; Smyth et al., 2003; Rendall et al., 2008; Suire et al., 2020). Studies which do report a sexual orientation difference in mean pitch are compromised by small sample sizes or lack of statistical tests: One such study, using voice samples from a debate between two participants, observed that the heterosexual participant had a higher mean pitch than the homosexual participant (Podesva et al., 2002). Another study of Portuguese speakers found that seven homosexual men had 11% higher average pitch and 43% higher pitch variability than seven heterosexual men, but did not conduct any statistical tests to confirm these differences (Barbuio and Paulino, 2021). One of the few studies reporting a pitch difference tested women participants only, showing that 34 homosexual women spoke in a lower pitch and used less pitch variation when compared to 68 heterosexual women (Van Borsel et al., 2013). Many of these studies used relatively low sample sizes, and statistical power or other limitations may have been a factor in any null results. Therefore, the possibility of a sexual orientation difference in mean (F0) pitch levels is explored in Hypothesis 1 of the present study.

There are additional frequency-related variables to consider: These are the formants, which are energy peaks at specific frequencies in the signal (F1–F4) which have been argued to “shape” the speaker’s voice profile through changes in articulatory settings during vowel production (i.e., they produce so-called “overtones” as the airflow in the vocal tract vibrates at different frequencies depending on shape and size of the vocal tract; Cavalcanti et al., 2021). Higher formants are argued to reflect more speaker specific settings and thus allow for better speaker discrimination (Cao and Dellwo, 2019). In comparison with overall mean pitch (F0), there is more evidence of an effect of sexual orientation on formants in both men and women, especially with regards to specific vowel sounds. For example, in sampled speeches from 103 men and women, homosexual men had a higher F1 and lower F2 in /ɒ/ (“dock”), higher F2 and lower F1 in /i/ (“sit”), and higher F2 and marginally higher F1 in /æ/ (“cat”) than heterosexual men. Neutral observers then rated their speech on a scale from “sounds totally straight” to “sounds totally gay/lesbian,” and were able to predict the speaker’s sexual orientation with “significant success” (Pierrehumbert et al., 2004). It is therefore possible that differences in formant frequencies are partially responsible for perception of sexual orientation differences in speech patterns. In other studies, differences were found in F1 of /ɛ/ (“bed”) and F2 of /oʊ/ (“go”) in homosexual women and F1 of /ɛ/ and /æ/ in homosexual men, as compared to heterosexuals of the same sex (Munson et al., 2006). Higher F4 in /æ/ and higher F2 in /oʊ/ were reported for homosexual men, as well as lower F2 in /ɛ/, lower F1 and F4 in /æ/, and lower F1 in /oʊ/ for homosexual women (Rendall et al., 2008). A later study found homosexual males produced higher F1 of /ɛ/ and higher F2 of /æ/ and /ɛ/, but participants were speakers of German and Italian, respectively (Sulpizio et al., 2015). The present study examines the prospect of sexual orientation differences in formant frequencies in Hypothesis 2.

There is also the possibility of sexual orientation differences in other speech properties. Jitter is an indicator of vocal “roughness” or voice quality and measured as frequency irregularities (caused by irregular vocal fold vibration). Roughness measures are generally lower in women than in men (Brockmann et al., 2008; Ambreen et al., 2019). One study to date investigated sexual orientation differences in jitter, finding no difference between heterosexual and homosexual men, but did not make any comparisons in women (Suire et al., 2020). Cepstral peak prominence has been used as an alternative voice quality measure (e.g., acoustic measure of dysphonia) as it can be calculated from running speech. High CPP values indicate that a voice sounds harmonic (i.e., contains a periodic signal), while lower CPP values are associated with disturbed periodicity of the signal (see Watts et al., 2017). The present study will examine the possibility of sexual orientation differences in jitter and cepstral peak prominence in Hypothesis 3.

Another potential difference could be found in shimmer (irregularities in amplitude) or harmonics-to-noise ratio (HNR). Both of these variables have been used to measure vocal “breathiness,” which is typically higher in female voices (Van Borsel et al., 2009) and is associated with voice attractiveness in both sexes (Xu et al., 2013). Homosexual men appeared to have breathier voices than heterosexual men; but no comparisons were made in women (Suire et al., 2020). The present study explored shimmer and HNR in Hypothesis 4.

Additionally, there is a possible sexual orientation difference in rate of speech: a homosexual male participant was reported to have a slower rate of speech than the heterosexual male participant (Podesva et al., 2002), and a slower speech rate was perceived as “more gay” by listeners (Sulpizio et al., 2015). The present study examines the relationship between speech rate and sexual orientation in Hypothesis 5.

In summary, some but not all past findings suggest that speech and voice production could differ, on average, according to a speakers’ sexual orientation. However, one major limitation with past studies is that they rarely include a comparable female sample. Some previous studies only include male participants (Gaudio, 1994; Podesva et al., 2002), and many of those that do include women in the sample have other limitations such as testing fewer females than males (Waksler, 2001), homosexual women being excluded despite sampling homosexual men (Suire et al., 2020), bisexual women substantially outnumbering homosexual women in the “non-straight” group (Rendall et al., 2008), or lacking statistical tests entirely (Moonwomon-Baird, 1997). By including a large sample of women of varying sexual orientations, we hope to fill a gap in the literature with the present study.

Furthermore, past work frequently relied on recited text—a common approach to ensure that participants produce the exact sounds needed for analyses is to ask them to read a pre-defined statement or list of sounds (Crist, 1997; Linville, 1998; Rendall et al., 2005; Munson et al., 2006; Sulpizio et al., 2015; Suire et al., 2020; Barbuio and Paulino, 2021). However, a scientifically-created non-emotional text intended to showcase a range of phonemes resulted in significantly more male speakers being judged to “sound gay” compared to the sound of the same men when producing a dramatic passage or natural speech (Smyth et al., 2003). This raises the possibility that pre-determined text could seriously distort true cues of sexual orientation. It thus needs to be determined how well sexual orientation is assessed when speech is naturally produced, and the present study seeks to achieve this.

Finally, past studies have applied a narrow view on how participants are grouped—some studies take the approach of recruiting both bisexual and homosexual participants, then grouping them together into a single “homosexual” group for comparison with heterosexual participants (Pierrehumbert et al., 2004; Munson et al., 2006; Rendall et al., 2008). This is problematic, as other research suggests that bisexuals can be intermediate between heterosexual and homosexual individuals in some aspects of gender nonconformity, but closer to homosexual individuals in others (Rieger et al., 2020a). Furthermore, separate lines of research suggest that viewing sexual orientation as a continuum is more appropriate than viewing it as categories (Epstein et al., 2012; Savin-Williams, 2014), and that sexual orientation in women in particular might be more fluid than the same in men (Kinnish et al., 2005; Diamond, 2016). It is therefore possible that treating sexual orientation as a continuous variable might reveal more information about the relationship between sexual orientation and voice properties.

Full details on both the sample sizes and nature of the stimuli in reviewed studies can be found in Table 1. To address aforementioned limitations, the present study used a large data set of both men and women of varying sexual orientations (317 participants), with data consisting entirely of natural speech. We also aimed to capture the full range of differences between individuals by treating sexual orientation as a continuous variable throughout. As such, although we frame these predictions as comparing heterosexual and homosexual individuals, in each case, we expected, based on other work on gender nonconformity, that bisexual individuals are either intermediate between the two, or closer to one of the other groups than the other. Based on the reviewed literature, the following hypotheses were tested:

1. Overall mean F0, F0 range and spectral tilt will be higher in homosexual men than heterosexual men, and lower in homosexual than heterosexual women.
2. Formant frequencies (F1–F4) of the vowels /ɛ/, /æ/ and /oʊ/ will be higher in homosexual men than heterosexual men, and lower in homosexual than heterosexual women.
3. Jitter will be lower, and Cepstral Peak Prominence (CPP) will be higher in homosexual men than in heterosexual men. Conversely, Jitter will be higher, and Cepstral Peak Prominence (CPP) will be lower in homosexual women than in heterosexual women.
4. Shimmer will be significantly higher and HNR significantly lower in homosexual men than heterosexual men. Conversely, shimmer will be significantly lower and HNR significantly higher in homosexual than heterosexual women.
5. Homosexual men will have a slower speech rate than heterosexual men, and homosexual women will have a faster speech rate than heterosexual women.

**Table 1:** Association between sexual orientation and speech properties across 10 studies.

Study	Speaker sample	Speech type	Findings	H
Gaudio (1994)	4 heterosexual men, 4 homosexual men	Recited (fixed texts)	Pitch range and variability did not predict sexual orientation (SO)	1
Moonwomon-Baird (1997)	2 heterosexual women, 2 homosexual women	Natural (conversations)	Heterosexual women had higher pitches and greater pitch range No statistical tests performed	1
Waksler (2001)	12 heterosexual women, 12 homosexual women	Natural (explaining film plot)	No significant difference in pitch range or variability	1
Podesva et al. (2002)	1 heterosexual man, 1 homosexual man	Natural (long-duration debate)	Heterosexual speaker had significantly higher pitch Homosexual speaker had slower speech rate No significant difference in pitch range	1, 4
Pierrehumbert et al. (2004)	26 heterosexual men, 29 homosexual men, 16 heterosexual women, 16 bisexual women, 16 homosexual women	Recited (specific sentences)	Overall F1 and F2 values significantly lower in homosexual and bisexual women than heterosexual women F1 of /æ/ significantly higher and F2 of /æ/ marginally higher in homosexual men than heterosexual men	2
Munson et al. (2006)	11 heterosexual men, 11 bi/homosexual men, 11 heterosexual women, 11 bi/homosexual women	Recited (lists of words containing specific phonemes)	No significant overall effect SO on mean pitch or pitch range, spectral tilt, F1 or F2 F1 of /ɛ/ and F2 of /oʊ/ significantly differed in homosexual women, and F1 of /ɛ/ and /æ/ differed in homosexual men	1, 2
Rendall et al. (2008)	34 heterosexual men, 29 bi/homosexual men, 33 heterosexual women, 29 bi/homosexual women	Recited (lists of words, short sentences)	No significant overall effect of SO on mean pitch or pitch range, nor formant frequencies in men Mean F1 and F2 lower in bi/homosexual women compared to heterosexual women In homosexual/bi men, F4 higher in /æ/ and F2 higher in /oʊ/; in bi/homosexual women, F2 significantly lower and more variable in /ɛ/, F1 and F4 significantly lower and more variable in /æ/, F1 significantly lower and more variable in /oʊ/	2
Sulpizio et al. (2015)	16 heterosexual men, 16 homosexual men	Recited (specific German/Italian sentences)	German: Homosexual males produced significantly higher F1 in /ɛ/	2, 4
Suire et al. (2020)	48 heterosexual men, 58 homosexual men, 54 heterosexual women	Natural (re-telling story in own words in French)	French: No significant effect of SO on mean pitch or Jitter in men French: Mean pitch variation and harmonics-to-noise ratio significantly higher in homosexual men	1, 3
Barbuio and Paulino (2021)	7 heterosexual men, 7 homosexual men	Recited (specific passages in Portuguese)	Portuguese: Mean pitch 11% higher in homosexual men than heterosexual men Portuguese: Average pitch variability 43% higher in homosexual men than heterosexual men (14% higher peaks, 12% lower valleys). No statistical tests performed	1

Information is given on speaker sample size and demographics, the type of speech stimuli used, the basic findings (with regards to the present study’s dataset), and the hypothesis it corresponds with.

## Method

### Participants

Participants were 142 men and 175 women of varied sexual orientations recruited through a combination of pride festivals, university fairs, university mailing lists, and advertisements in LGBT magazines. The 142 recruited men self-identified as “exclusively straight” (*n* = 60), “mostly straight” (*n* = 11), “bisexual leaning straight” (*n* = 14), “bisexual” (*n* = 9), “bisexual leaning gay” (*n* = 4), “mostly gay” (*n* = 12), or “exclusively gay” (*n* = 32). The 175 women self-identified as “exclusively straight” (*n* = 40), “mostly straight” (*n* = 37), “bisexual leaning straight” (*n* = 12), “bisexual” (*n* = 16), “bisexual leaning lesbian” (*n* = 11), “mostly lesbian” (*n* = 23), or “exclusively lesbian” (*n* = 36). The mean (SD) age was 24.33 (8.43) for men and 24.39 (7.33) for women. For men, 82% were White, followed by 5% Chinese, 3% Indian, and other ethnicities. For women, 78% were White, 5% Chinese, 5% African, and other ethnicities.

Participants reported their sexual orientation (as aforementioned) and attraction to men or women on 7-point scales (Kinsey et al., 1948). These two scales were highly correlated in both men, *p* < 0.0001, *r* = 0.98, 95% CI [0.96, 1.00], and women, *p* < 0.0001, *r* = 0.97 [0.93, 1.00], and were averaged within participants. For this sexual orientation average, a score of 0 represented exclusive heterosexuality, a score of 3 bisexuality with equal attractions, and 6 represented exclusive homosexuality.

### Procedure

#### Interview session

The University of Essex’s Ethics Committee approved this study (GR1303), and all experimental procedures were performed in accordance with the relevant guidelines and regulations. After providing written informed consent, participants completed a survey on their demographics and sexual orientation. Their voice was recorded using a Panasonic HDC-SD5 camera as part of an interview lasting 5–10 min. Questions were asked about the weather, their interests, and their childhood, and participants were not interrupted while answering. Participants were compensated monetarily for their time.

#### Audio data segmentation

For analyses, we extracted each participant’s full answer to the question “How would you describe the weather in England at this time of year to someone who had never been here before?” This question was selected because its content was engaging but not personal, and because it prompted most participants to give detailed answers. Recordings were extracted from the camera in their raw MTS file format. Using Audacity 2.3.2, the section of the audio containing their answer was then extracted into 32-bit float PCM WAV format files.

The resulting 317 audio files were then processed in Praat 6.0.55 (Boersma and Weenink, 2019). The audio files were first split into chunks containing phrases or sentences, and transcribed. The phrase/sentences (from now on referred to “utterance”) data were used for all non-vowel analyses. Next, three target vowels were identified throughout speech samples: /ɛ/ (as in “bed”), /æ/ (as in “cat”) and /oʊ/ (as in “go”). Data of each participant were limited to one instance of each vowel, with instances being at least 50 ms in duration. In cases where the vowel was produced multiple times, we selected the clearest example of it for extraction. Not all participants produced all vowels, resulting in a slightly imbalanced sample of vowels available for analyses. In total, 227 instances of /ɛ/, 128 instances of /æ/ and 172 instances of /oʊ/ were extracted, and the four formant frequencies (F1–F4) of these three vowels were averaged separately for analyses, resulting in a total of 294 participants for whom vowel data were available.

### Acoustic analyses and variables

Once labeled, individual utterances and vowels were extracted for each participant using automated scripts in Praat (Boersma and Weenink, 2019). Acoustic analyses were conducted in VoiceLab (Feinberg and Cook, 2020) with default settings. We chose VoiceLab because the software allows to automatise acoustic analyses, plus, does not require *a priori* knowledge of the speakers’ gender. This allows the data to be processed without manually specifying pitch floors and ceilings by gender, which could cause loss of high and low pitch data, and which we considered crucial to avoid as we were looking for gender-nonconforming voice properties. Analyses are reproducible by those without knowledge in acoustics (Feinberg and Cook, 2020). Unless otherwise stated, all variables were averaged across every utterance a participant gave prior to analysis.

### Pitch frequency

Mean F0 represents the mean pitch frequency of an utterance caused by the frequency of the vibration of the vocal folds, and is perceived by listeners as the “overall” pitch of an individual’s voice. Pitch Range represents the difference in pitch between a participant’s average maximum F0 and average minimum F0 across all utterances.

For three male participants, the software returned a mean pitch that was several standard deviations higher than all other participants, potentially because of external noise in the recording. This only applied to their utterance and not to the vowel data. These males were thus excluded from all pitch analyses of utterances (i.e., all of Hypothesis 1) as well as the pitch-related descriptive statistics in Table 2, but were included in all other analyses as normal. Including or excluding these 3 participants did not change the direction, strength or significance of any pitch-related analysis.

**Table 2:** Mean (standard deviation) of all speech variables across all participants.

	Males			Females		
Variable	Hetero-sexual	Bisexual	Homo-sexual	Hetero-sexual	Bisexual	Homo-sexual
Mean F0 (Hz)	131.5 (28.3)	117.8 (22.2)	117.5 (22.2)	196.8 (27.3)	198.0 (24.4)	192.7 (28.1)
Pitch range (Hz)	92.7 (58.3)	72.0 (52.3)	58.24 (40.0)	114.5 (54.9)	125.5 (55.5)	115.0 (70.1)
Spectral tilt	−0.37 (0.02)	−0.38 (0.03)	−0.37 (0.02)	−0.37 (0.02)	−0.36 (0.03)	−0.35 (0.02)
F1 mean (Hz)	624.4 (123.6)	617.9 (74.3)	599.78 (87.7)	687.9 (107.6)	684.5 (124.3)	668.5 (97.5)
F2 mean (Hz)	1562.5 (273.6)	1478.9 (251.1)	1572.7 (196.9)	1755.5 (246.4)	1747.1 (264.1)	1747.1 (250.7)
F3 mean (Hz)	2681.0 (386.5)	2671.5 (287.4)	2639.4 (276.6)	2889.0 (345.4)	2959.5 (393.7)	2807.8 (249.5)
F4 mean (Hz)	3766.6 (619.4)	3729.0 (361.5)	3702.1 (352.4)	4083.8 (429.9)	4120.7 (478.2)	3927.2 (303.1)
Jitter (local)	0.028 (0.009)	0.027 (0.013)	0.027 (0.008)	0.022 (0.007)	0.025 (0.008)	0.025 (0.006)
Jitter (RAP)	0.016 (0.005)	0.015 (0.008)	0.015 (0.006)	0.012 (0.004)	0.014 (0.005)	0.013 (0.003)
Cepstral peak prominence	25.27 (1.82)	24.45 (2.38)	25.11 (1.40)	26.82 (2.02)	25.89 (2.59)	26.58 (2.11)
Shimmer (local)	0.193 (0.070)	0.187 (0.057)	0.169 (0.049)	0.163 (0.045)	0.166 (0.067)	0.167 (0.039)
Shimmer (APQ3)	0.097 (0.054)	0.092 (0.037)	0.085 (0.029)	0.074 (0.023)	0.076 (0.046)	0.079 (0.037)
Harmonics-to-noise ratio	3.98 (1.49)	3.48 (1.09)	3.48 (1.25)	5.78 (1.76)	5.28 (1.86)	6.04 (1.45)
Speech rate	4.60 (0.97)	4.64 (0.75)	4.59 (0.81)	4.32 (0.80)	4.40 (0.95)	4.38 (0.86)

Participants were grouped according to their scores on the Kinsey scale, with Kinsey 0–1 considered heterosexual, 2–4 considered bisexual and 5–6 considered homosexual.

### Spectral tilt

Spectral Tilt represents the ratio of high frequency to low frequency sounds in the voice, and is measured by fitting a regression line to the frequency spectrum which makes up a sound, with steeper negative regression lines indicating a “noisier,” or breathier, voice sample and positive regression indicating “creakier” voice (Owren and Bachorowski, 2007). Specifically, these were calculated by comparing the maximum amplitudes within each frequency region around the first two harmonic frequencies and the first three formant frequencies, then examining the differences between these maxima (Boersma and Weenink, 2019).

### Formant frequencies

After the vocal folds generate a sound, it travels up the vocal tract and is filtered by the larynx. This filtration attenuates certain frequencies of sound and passes others, producing four distinct energy “peaks” in the frequency curve of a vowel sound. These four peaks are known as vowel formants (F1–F4), and make up the constituent parts of a vowel sound (Rendall et al., 2008). These formants are extracted automatically from vowel sounds by Praat and VoiceLab (Boersma and Weenink, 2019; Feinberg and Cook, 2020).

### Jitter and shimmer

Jitter and shimmer represent the “roughness” of a voice, and are related to the cycle-to-cycle variability of the mean fundamental frequency (F0) for phrases and vowels, respectively (Brockmann et al., 2008). Since they are related to F0, their absolute values, local shimmer and local jitter, are affected by the magnitude of F0. The present study thus also includes additional jitter and shimmer variables (RAP jitter and APQ3 shimmer), which are standardized to adjust for differences in F0 between speakers. Specifically, RAP jitter represents “the average absolute difference between a period and the average of it and its two neighbors, divided by the average period,” and APQ3 shimmer represents “the average absolute difference between the amplitude of a period and the average of the amplitudes of its three neighbors, divided by the average amplitude” (Boersma and Weenink, 2019).

### Cepstral peak prominence

Vocal roughness or breathiness (sometimes called “quality”) are often measured in a medical context to diagnose problems with the larynx. For this purpose, older studies have relied on measurements of jitter, shimmer, or harmonics-to-noise ratio (Heman-Ackah et al., 2002), with the latter two being the only variables previously studied with regards to sexual orientation (Suire et al., 2020). However, there is some indication that Cepstral Peak Prominence (CPP) may be a more comprehensive measure of voice quality (Fraile and Godino-Llorente, 2014). The present study therefore includes CPP as an exploratory variable. Praat and VoiceLab measure CPP as “difference in amplitude between the cepstral peak and the corresponding value on the trend line that is directly below the peak (i.e., the predicted magnitude for the quefrency at the cepstral peak)” (Boersma and Weenink, 2019).

### Harmonics-to-noise ratio

Harmonics-to-noise ratio is perceived as the “breathiness” or roughness of a voice, and represents the ratio between “periodic” and “non-periodic” components—the periodic component being the vibration of the vocal cords, and the non-periodic component being glottal noise. Thus, a higher ratio (meaning a greater amount of vocal cord vibration relative to glottal noise) means a higher-quality voice sound (Teixeira et al., 2013). Harmonics-to-noise ratio is calculated as “dB: if 99% of the energy of the signal is in the periodic part, and 1% is noise, the HNR is 10*log10(99/1) = 20 dB. A HNR of 0 dB means that there is equal energy in the harmonics and in the noise” (Boersma and Weenink, 2019).

### Speech rate

Speech rate was calculated by counting the number of syllables per utterance of a participant, then dividing this by the total duration of that utterance. Speech rate for each single utterance was then averaged across all utterances of a speaker.

## Results

Although we treat sexual orientation as a continuous variable in all analyses, we first present a summary of our key variables with participants grouped according to their scores on the Kinsey scale, with Kinsey 0–1 considered heterosexual, 2–4 considered bisexual, and 5–6 considered homosexual (Table 2). We also begin analyses with an examination of sex differences across all participants, regardless of sexual orientation, in order to verify that our data processing was successful.

Of the 32 conducted analyses, 8 returned significant differences that included the factor sexual orientation. For the sake of brevity, we do not report specific statistics for non-significant relationships in the following analyses. Instead, a full summary of all sexual orientation analyses in both sexes, including non-significant results, can be found in Table 3. Across all analyses, we tested for both a linear and curvilinear effect, to account for the possibility that bisexual individuals might be closer to heterosexual or homosexual individuals in some variables (Rieger et al., 2020a). However, none of these curvilinear effects were statistically significant, and so the following analyses focus on linear effects exclusively. Furthermore, for the sake of simplicity, our commentary focuses on comparing heterosexual to homosexual participants but, given the nature of using sexual orientation as a continuous variable, the implication is that bisexual individuals were intermediate between heterosexual and homosexual. Had this not been the case for any given variable, we would have found it in the form of a significant curvilinear effect.

**Table 3:** Linear regression analyses for voice properties predicted by sexual orientation in male and female participants.

	Males			Females			
Variable	*N*	*β* [95% CI]	*p*	*N*	*β* [95% CI]	*p*	H
Mean pitch (F0)	139	−0.25 [−0.41, −0.09]**	0.003	175	−0.11 [−0.26, 0.04]	0.137	1−
Pitch range	139	−0.28 [−0.44, −0.12]**	0.001	175	0.00 [−0.15, 0.15]	0.961	1−
Spectral tilt	139	−0.04 [−0.21, 0.13]	0.612	175	0.20 [0.05, 0.35]**	0.008	1+
F1 mean	124	−0.09 [−0.27, 0.09]	0.327	155	−0.11 [−0.27, 0.05]	0.173	2
F2 mean	124	0.02 [−0.16, 0.20]	0.810	155	−0.03 [−0.19, 0.13]	0.709	2
F3 mean	124	−0.05 [−0.23, 0.13]	0.562	155	−0.10 [−0.26, 0.05]	0.194	2
F4 mean	124	−0.06 [−0.24, 0.12]	0.510	155	−0.16 [−0.32, −0.01]*	0.041	2+
Jitter (local)	142	−0.04 [−0.20, 0.13]	0.672	175	0.16 [0.01, 0.31]*	0.036	3+
Jitter (RAP)	142	−0.04 [−0.41, 0.34]	0.850	175	0.16 [0.01, 0.31]*	0.035	3+
Cepstral peak prominence	142	−0.04 [−0.21, 0.12]	0.608	175	−0.07 [−0.22, 0.08]	0.365	3
Shimmer (local)	82	−0.12 [−0.34, 0.10]	0.286	104	0.02 [−0.17, 0.22]	0.814	4
Shimmer (APQ3)	80	−0.07 [−0.30, 0.15]	0.530	104	0.06 [−0.14, 0.25]	0.562	4
Harmonics-to-noise ratio	142	−0.19 [−0.35, −0.02]*	0.025	175	0.03 [−0.12, 0.18]	0.741	4-
Speech rate	142	−0.02 [−0.19, 0.15]	0.81	175	0.04 [−0.11, 0.19]	0.576	5

A positive standardized regression coefficient (β) indicates that scores in a given variable are higher in homosexual participants than heterosexual participants. Numbers in brackets represent 95% confidence intervals of the standardized regression coefficient, β. H denotes the hypothesis the variable corresponds with, and + or – in the H column denotes whether this effect was in line with predictions. *p < 0.05, **p < 0.01.

### Sex differences

In order to verify that the steps taken in our data processing aligned with previous results, we first checked for expected sex differences across all variables shown in Table 2. Since these are outside of the scope of our research, we do not report them in full here. All of the variables reported in this paper returned significant sex differences in the expected directions. For these sex differences, effect sizes (*Cohen’s d*) ranged between 0.23 and 2.67, with a median of 0.63; *p-values* for associated t-tests ranged from 0.0001 to 0.0437, with a median of 0.0001. Thus, we are satisfied that the steps taken during data processing and analysis were successful and our data set aligned with past work. We conclude from this that the statistical tests reported here represent genuine natural speech differences between speakers of different sexual orientation.

### Hypothesis 1

We hypothesized that overall mean F0, F0 range and spectral tilt would be higher in homosexual men than heterosexual men, and lower in homosexual women than heterosexual women. For both men and women, we separately regressed the variables mean F0, F0 range, and spectral tilt onto sexual orientation.

**Figure 1:**
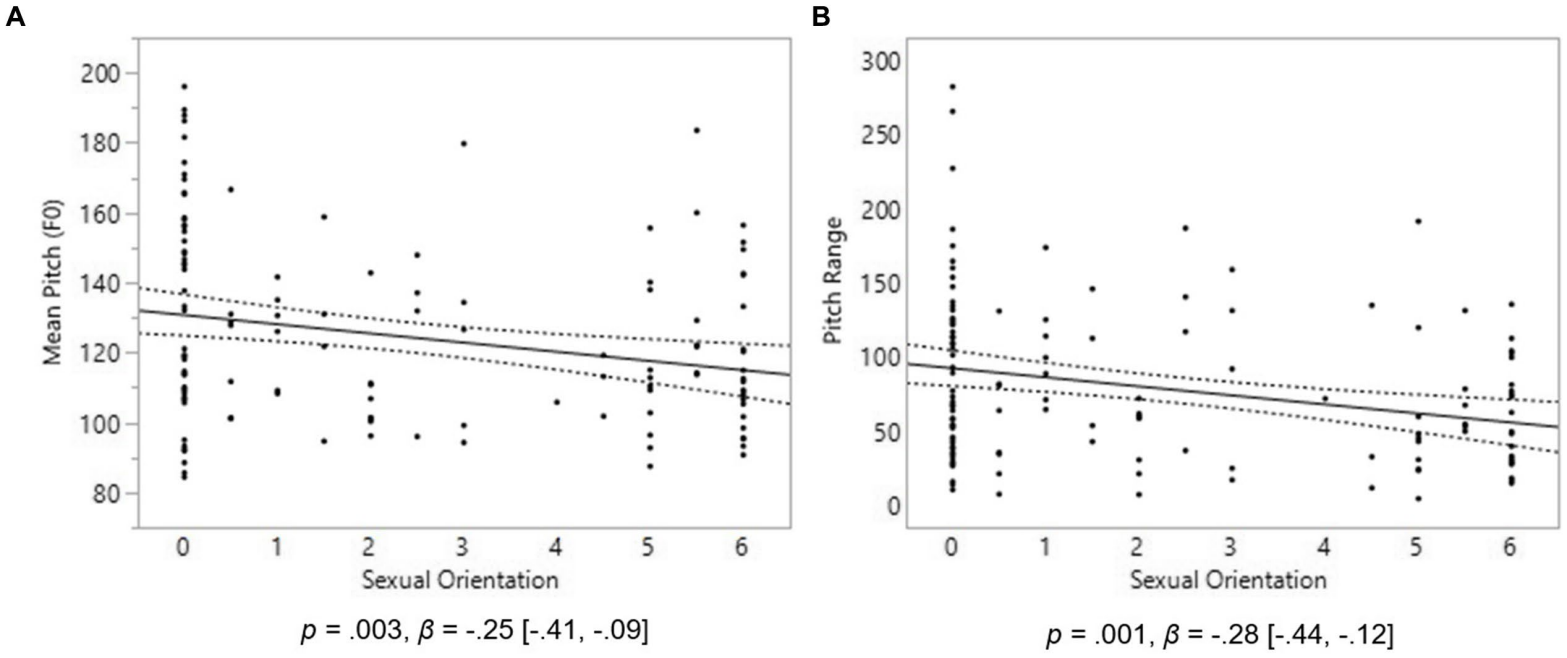
Voice pitch properties in 139 males. On the Y axes are Mean Voice Pitch (F0) **(A)** and Pitch Range **(B)**. On the X axis, 0 represents exclusive heterosexuality, 3 bisexuality, and 6 represents exclusive homosexuality. Triple lines represent regression coefficients with their 95% confidence intervals. Dots represent participants’ average scores.

In males, a significant relationship was found between sexual orientation and mean F0; however, contrary to our hypothesis, homosexual males had lower-pitched voices than heterosexual males, *p* = 0.003, *β* = −0.25, 95% CI [−0.41, −0.09] (Figure 1A). Similarly, the F0 range values were significantly lower in homosexual men, *p* = 0.001, *β* = −0.28, [−0.44, −0.12] (Figure 1B), suggesting a restricted pitch use while answering our question. This finding was also in contrast to our prediction. No relationship was found between sexual orientation and spectral tilt in males.

**Figure 2:**
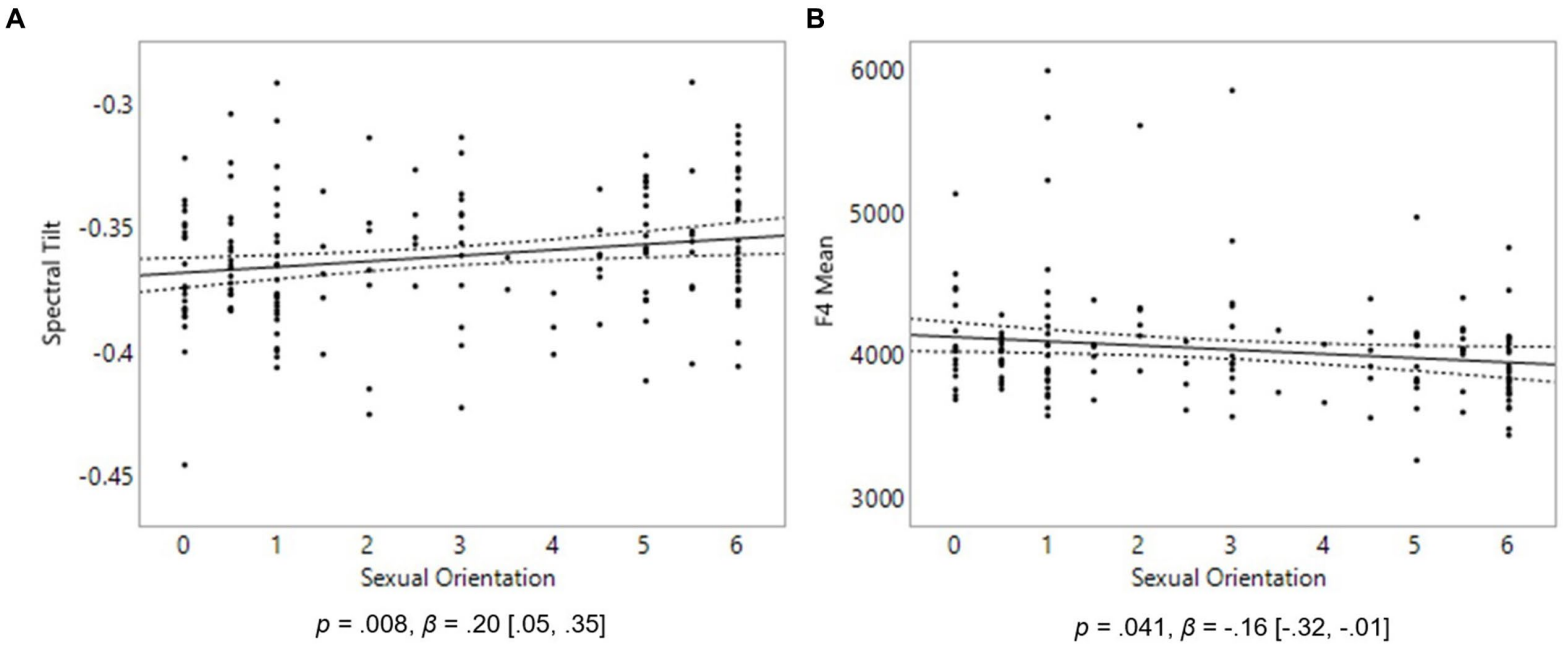
Voice properties in 175 females. On the Y axes are Spectral Tilt **(A)** and F4 Mean **(B)**. On the X axis, 0 represents exclusive heterosexuality, 3 bisexuality, and 6 represents exclusive homosexuality. Triple lines represent regression coefficients with their 95% confidence intervals. Dots represent participants’ average scores.

In females, the only statistically significant finding was that homosexual women’s spectral tilt was not as steep as that extracted from heterosexual women, *p* = 0.008, *β* = 0.20, [0.05, 0.35] (Figure 2A). Consistent with our predictions, this indicates that homosexual women had a less breathy voice than heterosexual women, on average. No relationship was found between sexual orientation and mean pitch or pitch range in females.

### Hypothesis 2

We hypothesized that formant frequencies (F1–F4) of the vowels /ɛ/, /æ/ and /oʊ/ would be higher in homosexual men than heterosexual men, and lower in homosexual women than heterosexual women. No significant relationship was found between sexual orientation and any of the four formant frequencies in males. In females, homosexual women had a lower F4 than heterosexual women, as predicted, *p* = 0.041, *β* = −0.16, [−0.32, −0.01] (Figure 2B). No significant relationship was found between sexual orientation and any of the other three formant frequencies in females.

### Hypothesis 3

**Figure 3:**
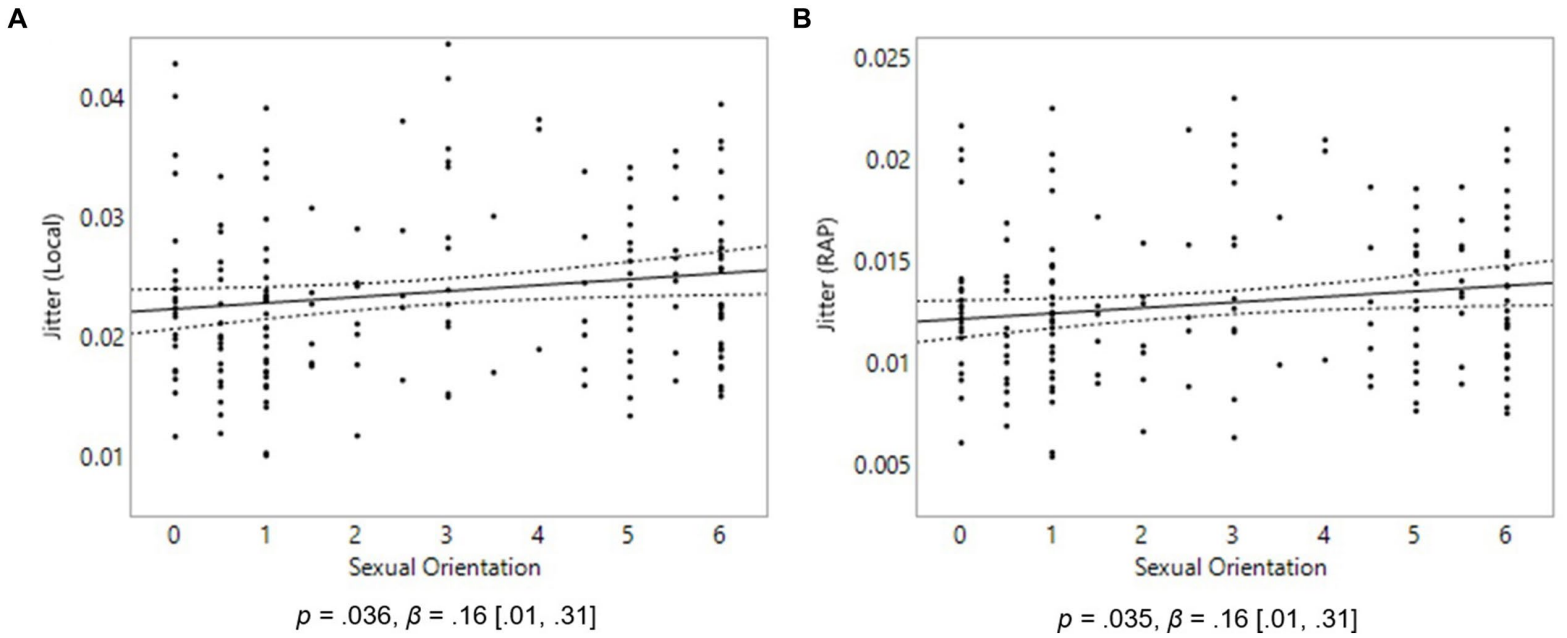
Voice properties in 175 females. On the Y axes are Local Jitter **(A)** and RAP Jitter **(B)**. On the X axis, 0 represents exclusive heterosexuality, 3 bisexuality, and 6 represents exclusive homosexuality. Triple lines represent regression coefficients with their 95% confidence intervals. Dots represent participants’ average scores.

We hypothesized that jitter would be lower, and cepstral peak prominence higher, in homosexual men compared to heterosexual men, we further hypothesized that jitter would be higher, and cepstral peak prominence lower, in homosexual women compared to heterosexual women. In males, no relationship was found between sexual orientation and any of the variables *local jitter*, *RAP jitter*, or *cepstral peak prominence*. In females, in line with our hypotheses, homosexual women in the sample displayed more jitter in their voice than heterosexual women when looking at both *local jitter* and *RAP jitter* variables, *p* = 0.036, *β* = 0.16, [0.01 0.31], Figure 3A and *p* = 0.035, *β* = 0.16, [0.01, 0.31], Figure 3B, respectively. No relationship was found between sexual orientation and cepstral peak prominence in females.

### Hypothesis 4

**Figure 4:**
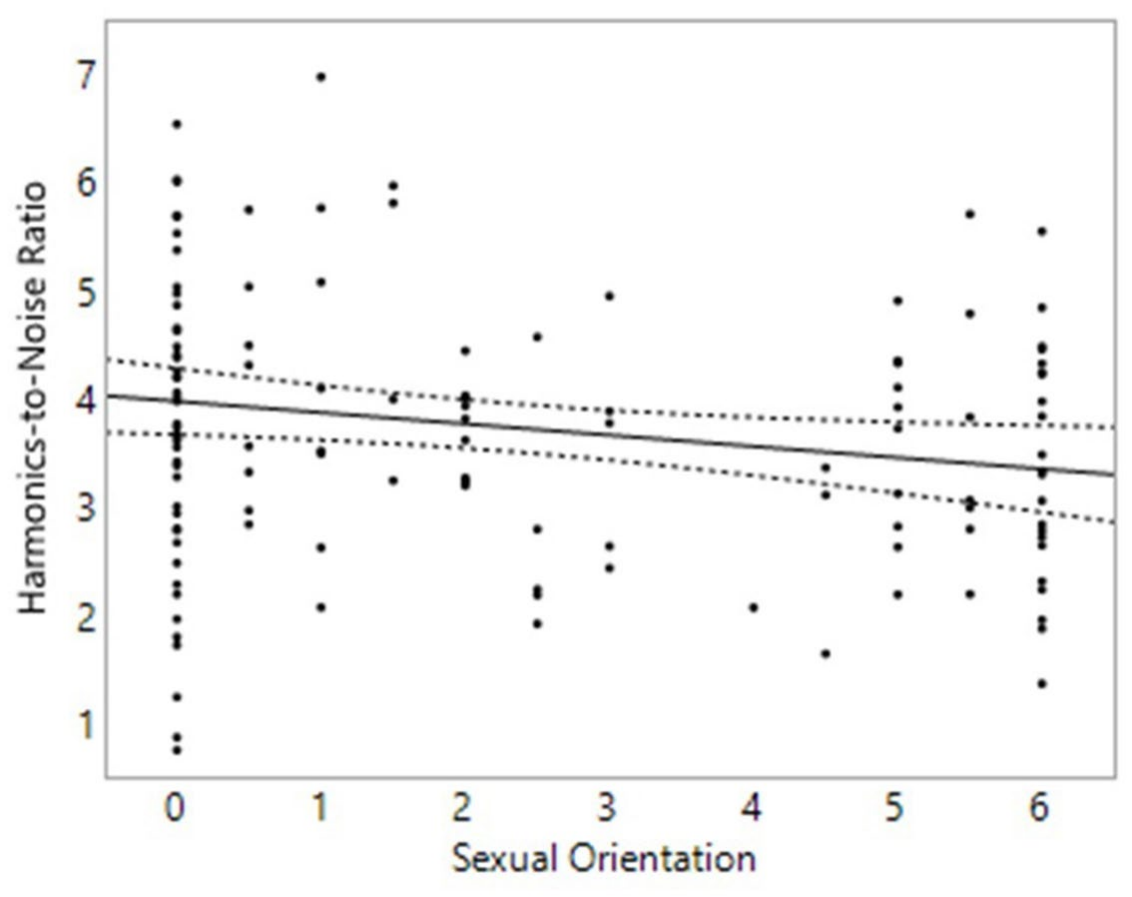
Voice properties in 142 males. On the Y axis is Harmonics-to-Noise Ratio, measured in dB. On the X axis, 0 represents exclusive heterosexuality, 3 bisexuality, and 6 represents exclusive homosexuality. Triple lines represent regression coefficients with their 95% confidence intervals. Dots represent participants’ average scores.

We hypothesized that shimmer measurements would be significantly higher in homosexual men than heterosexual men, and lower in homosexual women than heterosexual women. For harmonics-to-noise ratio (HNR), we expected a breathier voice (lower HNR) in homosexual men compared to heterosexual men and a breathier voice for heterosexual women compared to homosexual women. In males, contrary to our prediction, homosexual men had a lower HNR than heterosexual men on average, *p* = 0.025, *β* = −0.19, [−0.35, −0.02] (Figure 4). No relationship was found between sexual orientation and either local shimmer or APQ3 shimmer in males. In females, no relationship was found between sexual orientation and any of these variables.

### Hypothesis 5

We hypothesized that homosexual men would have a slower speech rate than heterosexual men, and homosexual women will have a faster speech rate than heterosexual women. No relationship was found between sexual orientation and speech rate in either males or females.

## Discussion

The current study set out to describe spectral profile characteristics of speakers from varying sexual orientations. In contrast to previous studies, we used only natural speech instead of relying on recited passages, and treated sexual orientation as a continuous variable, thereby making an important advancement in the literature around sexual orientation differences in acoustic properties. As fewer previous studies focused on women than men, many of our predictions for homosexual women were simply the opposite of findings in homosexual males, in line with gender nonconformity theory. In line with past findings, the present data confirm a range of sexual orientation differences in acoustic properties in both men and women, but also fail to replicate some past results. In the following, we discuss these findings separately for main acoustic features.

### Frequency related variables

Homosexual male speech is commonly stereotyped as being high-pitched; and even male television actors speak in a higher-pitched voice when playing homosexual characters (Cartei and Reby, 2012). However, as outlined, the majority of studies exploring this in larger samples do not confirm this difference (Munson et al., 2006; Rendall et al., 2008; Suire et al., 2020; Barbuio and Paulino, 2021). One study found that homosexual men had a lower pitch, but with only 2 participants and no statistical tests (Podesva et al., 2002). Similarly, numerous studies have not found a sexual orientation difference in pitch variability for men (Gaudio, 1994; Podesva et al., 2002; Rendall et al., 2008), although one study found that homosexual speakers of French had a greater pitch range (Suire et al., 2020), the opposite of the present findings. Examination of Mean F0 and F0 range in our large sample of speakers showed homosexual men speaking in a lower pitch with a narrower range than heterosexual men, a finding not in line with the view that homosexuals display more feminized voice characteristics. Given the mixed findings around pitch measurement, any interpretation of the current effects remain speculative. However, it should be noted that past research indicates that both men and women perceive low-pitched male voices as more attractive and relate them to positive personality traits (c.f., Feinberg et al., 2008; Tigue et al., 2012), and narrow pitch range use has also been linked to sounding more attractive (Xu et al., 2013). Group differences in terms of vocal pitch may therefore be explained by a speakers’ desire to sound more attractive, and future studies could monitor this to help account for the mixed findings.

This study also examined sexual orientation differences in formant frequencies (F1–F4), which have been found in previous research (e.g., Pierrehumbert et al., 2004; Munson et al., 2006). As predicted, homosexual women in our sample had a lower-frequency F4 formant than heterosexual women, and in line with previous research which investigated formant frequencies across all vowels, we found no effect in F1 or F2 (Munson et al., 2006). Although this technique reduced the number of statistical tests, it may have led to washing out effects for lower formants. Crucially, F4 has been linked to better speaker discrimination (Cao and Dellwo, 2019) and there is evidence to suggest that F4 is more prone to speaker variation than vowel production (Marrero et al., 2008) emphasizing that F4 might prove more useful for investigating sexual orientation effects in future research.

Finally, past research has investigated jitter, the irregularities in vocal fold vibration, which has been taken as an indicator of vocal roughness. Female voices are described as less rough, while male voices exhibit increased jitter values (c.f., Suire et al., 2020). In line with the gender nonconformity hypothesis (and with Hypothesis 3), the current data revealed that homosexual women had greater jitter values than heterosexual women, making their voice sound slightly rougher. No such difference was found in men either here or in a recent study (Suire et al., 2020); and as roughness is positively associated with vocal attractiveness in men (Hughes et al., 2004), the null finding contradicts our previous idea of homosexual men potentially aiming to sound more attractive. Still, future studies should continue to monitor this variable and should examine the impact of different vowel averaging techniques and their impact on findings.

### Spectral tilt

Past work has used spectral tilt (or slope) measurements as an indicator of voice quality. It has been argued that more energy in higher frequencies (i.e., a flatter spectral tilt) is an indication of a bright, resonant voice (LeAnn and Claire, 2022) and that an greater spectral tilt indicates more breathiness (Owren and Bachorowski, 2007). Here, we found that homosexual women had a flatter spectral tilt compared to heterosexual women, indicating a brighter voice quality. The only previous study investigating this did not find a relationship with sexual orientation in either sex (Munson et al., 2006), but a recent study comparing non-binary (assigned female at birth) and cisgender participants report a less negative spectral tilt for non-binary participants (LeAnn and Claire, 2022).

### Harmonics-to-noise ratio

In men, homosexuals had a lower HNR (less “breathy” voices) than heterosexuals. This is the opposite pattern to the one observed previously in French male speakers (Suire et al., 2020), where homosexual men were found to have a higher harmonics-to-noise ratio than heterosexual men. The present study was the first of its kind to examine this relationship in a sample of English speakers, and it remains to be seen whether, and in what directions, sexual orientation differences in harmonics-to-noise ratio are reliable in larger samples across different languages.

### All other indicators

To build a complete picture of the effects of sexual orientation on acoustic properties, we tested a wide range of other indicators. Some past work reported sexual orientation effects on these other cues, but none of them were significant in the present dataset. Yet, expected sex differences typically reported for male speech when compared to female speech were present for almost every variable. Not only does this confirm reliability of our data, but—crucially—it suggests that sexually dimorphic acoustic features that help to differentiate male speech from female speech are more stable and reliable than the acoustic features which differentiate between sexual orientations. Indeed, it is clear from this large data set that not all acoustic properties relate to sexual orientation in a meaningful way in *either* sex, while others (e.g., mean pitch, harmonics-to-noise ratio, jitter) had a relationship with sexual orientation in one sex but not the other, though sometimes one contrary to past reports. It is possible that these variables simply do not relate to sexual orientation, and that differences to past findings are due to methodological differences between studies. Previous work has often used small sample sizes, highly controlled lab environments (e.g., single vowel production), and recited text. Recall that Smyth et al. (2003) reported differences in ratings from naïve listeners for how “gay” a voice sounded depending on the recording instruction. If the voice cues used to judge sexual orientation reflect objective differences in voice quality, it stands to reason that future research should include at least one natural speech condition, even if recited passages are also used to ensure consistent data availability between speakers.

### Limitations

Although the use of natural speech conferred benefits to this study, it is also true that it brought its own set of limitations. Specifically, allowing participants to say whatever they like produces more natural speech patterns, but it also restricts analyses because specific elements might not be available for analysis in each sample. In particular, we experienced problems with the formant frequency analysis, since the majority of produced vowels were not long enough in duration to be suitable for acoustic analysis. As a result, we decided to target only three types of vowel, as well as to isolate only the single vowel produced by each participants which was longest, and most suitable for analysis. This reduced the amount of data available for analysis significantly.

A final point, and another potential source of distortion in previous studies, is the growing body of evidence that the “gay voice” might be a method of social signaling: In other words, individuals may modulate their voice patterns intentionally to reveal or conceal their sexual orientation. Daniele et al. (2020) found that gay speakers were rated as sounding “more gay” when imagining they are speaking to an individual who reacted positively to their sexual orientation, as opposed to an individual who reacted badly or who was unaware of their sexual orientation. Furthermore, they found that YouTubers sounded “more gay” in videos recorded after they revealed their sexual orientation publicly. Again, assuming that differences in ratings reflect, to some degree, objective differences in speech patterns, this means results may differ when participants are aware that their voice samples are to be used in a study examining the impact of sexual orientation on speech properties. If our participants did not see a strong reason to signal their sexual orientation during the interview by changing voice patterns, this may have altered any potential relationship between voice properties and sexual orientation in the present dataset. It is difficult to judge whether this also affected past research, because many studies do not explicitly state whether or not participants were aware of the purpose of the voice study. Future researchers should therefore take care when deciding whether participants are to be made aware of the study objectives in advance of voice samples being collected, or could even conduct a study specifically aimed at comparing voice properties before and after participants are made aware that sexual orientation is the focus of the research.

## Data availability statement

The raw data supporting the conclusions of this article will be made available by the authors, without undue reservation.

## Ethics statement

The studies involving humans were approved by The University of Essex Ethics Committee (GR1303). The studies were conducted in accordance with the local legislation and institutional requirements. The participants provided their written informed consent to participate in this study.

## Author contributions

LH: Conceptualization, Data curation, Formal analysis, Investigation, Methodology, Project administration, Software, Validation, Visualization, Writing – original draft, Writing – review & editing. GR: Conceptualization, Data curation, Formal analysis, Funding acquisition, Investigation, Methodology, Supervision, Visualization, Writing – original draft, Writing – review & editing. SP: Conceptualization, Formal analysis, Investigation, Methodology, Software, Supervision, Validation, Writing – original draft, Writing – review & editing.
